# The association between street connectivity and depression among older Japanese adults: the JAGES longitudinal study

**DOI:** 10.1038/s41598-022-17650-w

**Published:** 2022-08-08

**Authors:** Yu-Ru Chen, Masamichi Hanazato, Chie Koga, Kazushige Ide, Katsunori Kondo

**Affiliations:** 1 Graduate School of Medical and Pharmaceutical Sciences, Chiba University, 1-8-1 Inohana, Chuo-ku, Chiba-shi, Chiba 260-8672 Japan; 2 Center for Preventive Medical Sciences, Chiba University, 1-33 Yayoi-cho, Inage-ku, Chiba-shi, Chiba 263-8522 Japan; 3 Department of Community General Support, Hasegawa Hospital, Yachimata-shi, Chiba 289-1103 Japan; 4 Center for Gerontology and Social Science, National Center for Geriatrics and Gerontology, 7-430 Morikoka-cho, Obu-shi, Aichi 474-8511 Japan

**Keywords:** Epidemiology, Geriatrics

## Abstract

Mental health is important in older age; neighborhood environment is considered a protective factor of depression. Research has established that a critical indicator of neighborhood environment, street connectivity, is related to older people's health. However, little is known about the relationship between street connectivity and depression. We examined the relationship between street connectivity and depression among older people. Using Japan Gerontological Evaluation Study 2013–2016, the target population comprised 24,141 independent older people without depression (Geriatric Depression Scale scores below 5) in 2013. The outcome variable was depression in 2016; the explanatory variable was street connectivity calculated by intersection density and space syntax within 800 m around the subject’s neighborhood in 2013. We used logistic regression analysis to calculate the odds ratio and 95% confidence interval for the new occurrence of depression among participants in 2016. This analysis demonstrated incidence of new depression after 3 years that is 17% and 14% lower among participations living in high-intersection density and high-street-connectivity areas, respectively, than those living in low-intersection density and low-street-connectivity areas. The association held after adjusting for physical activities and social interaction. Given the established connection between street connectivity and mental health, the findings can contribute to healthy urban planning.

## Introduction

Mental health and well-being are as important in older age as at any other time of life. In 2017, 264 million people worldwide experienced a mental disorder, and the estimate in Japan is the second highest in Asia at 5.06 million suffering from depressive disorders^1,2^. It is a super-aged society and a country with a high proportion of adults aged ≥ 75 years. Previous studies documented that this population is experiencing serious psychological distress, such that depression is a common cause of suicide in older people of both sexes in Japan^3^. Depression is also associated with increased mortality mainly associated with frailty among older men^4^. In a systematic review of the relationship between depression and frailty, depressed older adults were more likely to experience frailty than those without depression, specifically men^4,5^.

Depression is a complex interplay between individual and environmental traits and characteristics, where individual characteristics refer to social, psychological, and biological factors, such as childhood adversity, loss, and unemployment^2,6^. Among the older people, depression is associated with genetic risk, non-genetic biological factors, physical illness, anxiety, sleep disturbance, elder abuse, and social risk factors^7,8^. Environmental contributions are related to the neighborhoods, where communities of individuals and neighborhood environment characteristics, such as walkability, community facilities, transportation, types of greenness, and proximity to an elementary school, have been linked with depression among older people^5,9–11^. Previous studies have reported that the mechanisms of the association between neighborhood environment and mental health can be explaned by loneliness, socially supportive relationships, and physical activity^12–14^. Thus, the presence of neighborhood environments that provide for physical and social activities are protective factors against depression^7^.

Among the neighborhood environmental correlations of depression outcomes, walkability refers to whether the neighborhood environment encourages people to walk, where previous studies report on the relationships between walkability and health^15,16^. For instance, in a cross-sectional study in European countries, improving walkability decreased loneliness in older adults with depression^17^, whereas older people in Japan living in neighborhoods with good walkability reported low prevalence of knee and lower-back pain^18^. Walkability can be measured using several indicators, where the commonly used indicators in the research are land-use mix, residential density, and street connectivity^15,16^. According to a systematic review on neighborhood environment and depression in older adults, 39% of papers (n = 73) are related to urbanization population/residential density, whereas 19% are related to diverse access to/availability of destinations. Both are associated with depression. However, only 1% focused on connectivity and found no association with depression^9^. Another systematic review described three out of 13 studies on connectivity and found inconsistent results between street connectivity and depression due to the cross-sectional study design, which may be due to reverse causation and require further research^19^. Furthermore, among the walkability indices, urban designers can easily measure and plan street connectivity compared with land-use mix and residential density. Lotfi and Koohsari (2011) reported that the availability and comparability of land-use mix data may be related to the difficulty of collecting data^20^. For these reasons, we focused on street connectivity among the walkability indices.

The concept of street connectivity has received increased attention from researchers, planners, and planning authorities^21^.We propose that street connectivity may influence depression due to street connectivity, which has been found to be associated with physical activity, social interaction, and cognitive function among older people. Two longitudinal studies of American and Australian adults identified that street connectivity is positively associated with walking for transport^22,23^. Moreover, superior street connectivity can encourage walking behavior and contribute to the formation of social networks among adults, such as gaining more friends and meeting neighbors^24^. Following previous studies^25,26^, we considered that higher street connectivity, where small street blocks and more frequent intersections were created, enhanced the walking behavior, extended spaces for building development, and encouraged the creation of more spots where people may meet. Furthermore, in a previous study, older people from 11 European countries reported that high levels of physical activity at baseline is related to low rates of depression after two of observation years^27^. Further, social interaction was found to exert a protective effect on depression among older people, and it has been reported that older Japanese males who frequently meet with their friends experience lower rates of depression over a 4-year period^28^. Researchers in Singapore identified an association between street connectivity and the cognitive function of older adults^29^. Moreover, a longitudinal study conducted over a 2-year period in the United States demonstrated that high street connectivity was associated with small declines in attention among older people without mild Alzheimer's disease. Meanwhile, in a systematic review and meta-analysis of 29 longitudinal studies, associations between cognitive function and subsequent depression were significant^30^. However, in terms of the relationship between street connectivity and depression, we found inconsistent results among previous studies^25,31^, and no longitudinal studies were conducted. Findings from cross-sectional studies conducted in Singapore and China suggest the influence of street connectivity on depression in a negative association^25,31^. In contrast, Zhang et al. reported that connectivity is associated with greater increases in depressive symptoms among older Chinese people^32^. Another cross-sectional study found no significant difference between street connectivity and depression among older Australian men^33^. Moreover, previous studies have certain limitations. First, previous researchers commonly highlighted the disadvantage of the cross-sectional study design, because it does not help establish causal relationships, such as those between built environments and depression among older people^21,29,31–33^. Second, the sample sizes were limited due to the inclusion of only men^33^ or of a small population for generalization^25^. Third, the mechanisms explaining the associations between street connectivity and depression remain unclear.

Therefore, the current study aimed to examine the association between street connectivity and depression among a large group of older Japanese adults of both sexes in a 3-year follow-up longitudinal study. We tested the following hypothesis: high street connectivity is associated with a lower likelihood of depression among older adults.

## Results

Table 1 presents the chi-squared results for the study participants' characteristics. Among the 24,141 older adult participants, 9.5% (n = 2292) became depressed during the 3-year follow-up period, of whom 52.1% were women. The following characteristics were common among the respondents who reported depression onset: age ≥ 75 years, < 10 years of education, low income, married status: as a widowed, divorced or never married, living alone, not using a car, do not walk, going out, seeing friends regularly, and without social participation. Residents in areas with low street connectivity and low land value reported depression.

**Table 1:** Characteristics of the Study Participants Classified by Depression Status (n = 24,141).

Characteristics (Total numbers)	Depression				χ^2^
	No		Yes		*p*-Value
	No	%	No	%	
**Sex**					
Male (n = 11,711)	10,614	48.6	1097	47.9	0.514
Female (n = 12,430)	11,235	51.4	1195	52.1	
**Age group, years**					
65–69 (n = 8185)	7523	34.4	662	28.9	< 0.001
70–74 (n = 8272)	7542	34.5	730	31.8	
75–79 (n = 4815)	4325	19.8	490	21.4	
80–84 (n = 2175)	1891	8.7	284	12.4	
≥ 85 (n = 694)	568	2.6	126	5.5	
**Education level, years**^a^					
≤ 9 (n = 8672)	7675	35.1	997	43.5	< 0.001
≥ 10 (n = 15,223)	13,949	63.8	1274	55.6	
Missing (n = 246)	225	1.0	21	0.9	
**Equivalent household income, million yen**					
Low (< 2.00) (n = 8938)	7899	36.2	1,039	45.3	< 0.001
Middle (2.00–3.99) (n = 9108)	8374	38.3	734	32.0	
High (≥ 4.00) (n = 2994)	2814	12.9	180	7.9	
Missing (n = 3101)	2762	12.6	339	14.8	
**Marital status**					
Married (n = 18,734)	17,038	78	1696	74	< 0.001
Widowed (n = 4011)	3588	16.4	423	18.5	
Divorced (n = 605)	531	2.4	74	3.2	
Never married (n = 433)	378	1.7	55	2.4	
Other/missing (n = 358)	314	1.4	44	1.9	
**Living situation**^b^					
Lives with others (n = 20,768)	18,836	86.2	1932	84.3	0.023
Lives alone (n = 2261)	2011	9.2	250	10.9	
Missing (n = 1112)	1002	4.6	110	4.8	
**Driving status**^c^					
Not a car user (n = 4483)	3948	18.0	535	23.3	< 0.001
Car user (n = 19,609)	17,858	81.7	1751	76.4	
Missing (n = 49)	43	0.3	6	0.3	
**Years of residence**					
< 10 (n = 1429)	1268	5.8	161	7.0	0.133
10–19 (n = 2285)	2053	9.4	232	10.1	
20–29 (n = 2425)	2210	10.1	215	9.4	
30–39 (n = 4131)	3749	17.2	382	16.7	
40–49 (n = 5771)	5251	24.0	520	22.7	
≥ 50 (n = 7743)	6993	32.0	750	32.7	
Missing (n = 357)	325	1.5	32	1.4	
**Frequency of going out**^d^					
A few times per year (n = 344)	287	1.3	57	2.5	< 0.001
Weekly (n = 3984)	3488	16.0	496	21.6	
Daily (n = 19,602)	17,889	81.9	1713	74.7	
Missing (n = 211)	185	0.8	26	1.1	
**Duration of daily walking, min**^e^					
Low (< 30) (n = 4417)	3864	17.7	553	24.1	< 0.001
Moderate (30–59) (n = 8884)	8023	36.7	861	37.6	
High (≥ 60) (n = 10,577)	9725	44.5	852	37.2	
Missing (n = 263)	237	1.1	26	1.1	
**Frequency of seeing friends**					
Less than once per month (n = 5043)	4416	20.2	627	27.4	< 0.001
More than once per month (n = 18,447)	16,865	77.2	1,582	69.0	
Missing (n = 651)	568	2.6	83	3.6	
**Social participation**					
Less than onces per month (n = 8378)	7366	33.7	1012	44.0	< 0.001
More than once per month (n = 14,039)	12,962	59.3	1077	47.0	
Missing (n = 1724)	1521	7.0	203	9.0	
**Quintiles of population density, people/km**^2^					
< 3322 (n = 6104)	5485	25.1	619	27.0	0.088
3322–4528 (n = 6395)	5772	26.4	623	27.2	
4528–9213 (n = 5609)	5107	23.4	502	21.9	
> 9213 (n = 6033)	5485	25.1	548	23.9	
**Quintiles of land value, yen/m**^2^					
< 35,500 (n = 6123)	5475	25.1	648	28.3	0.009
35,500–65,100 (n = 5974)	5422	24.8	552	24.1	
65,100–144,000 (n = 6043)	5501	25.2	542	23.6	
> 144,000 (n = 6001)	5451	24.9	550	24.0	
**Tertiles of intersection density, intersections/km**^2^—800 m circular buffer					
Low (≤ 155) (n = 8112)	7275	33.3	837	36.5	0.001
Moderate (156–216) (n = 8041)	7273	33.3	768	33.5	
High (≥ 217) (n = 7988)	7301	33.4	687	29.9	
**Tertiles of space syntax connectivity, numbers**					
Low (< 2.7) (n = 8048)	7191	32.9	857	37.4	< 0.001
Moderate (2.7–3.0) (n = 8067)	7356	33.7	711	31.0	
High (≥ 3.0) (n = 8026)	7302	33.4	724	31.6	

χ^2^ chi-squared test.

^a^How many years of formal education have you completed? 1 = Less than 6 years; 2 = 6 to 9 years; 3 = 10 to 12 years; 4 = 13 years or more; 5 = Others.

^b^How many people are in your household, including yourself? Whom do you live with? Circle all that apply. Live by myself/Live with (1) spouse, (2) son(s), (3) daughter(s), (4) spouse(s) of child(ren), (5) grandchild(ren), (6) brother(s) or sister(s), and (7) Others.

^c^Car user includes both self-driving and someone else drives.

^d^How often do you go out (including to the field or immediate neighborhood, for shopping, to the hospital?) 1 . four times or more per week 2. Two or three times per week 3. Once per week 4. One to three times per month 5. Several times per year 6. Rarely.

^e^How long do you walk a day on average? 1 = Less than 30 min; 2 = 30–59 min; 3 = 60–89 min; 4 = 90 min or more.

Table 2 presents the odds ratios (ORs) and 95% confidence intervals (CIs) of Models 1–3 from the logistic regression analysis for the association between street connectivity (intersection density and space syntax connectivity) and depression (the results of the full models are available in the Supplementary materials). For intersection density, in Model 1, we found a significantly negative association between an area with high-intersection density (OR = 0.83, 95% CI: 0.72–0.96) and depression onset controlled for demographic and socioeconomic covariates during the follow-up period. After adjusting for the factors of physical activities in Model 2 and adding social interaction as covariates in Model 3, the results of depression onset demonstrated a resemblance to model 1, which are both significantly negatively related to high-intersection density. Regarding space syntax connectivity, our results reveal that older adults living in areas with moderate (0.85, 0.76–0.95) and high (0.86, 0.75–0.97) street connectivity were 15% and 14%, respectively, less likely to report depression onset than those living in areas with low connectivity in Model 1. After adding physical activities in Model 2 and social interaction in Model 3, the results of depression onset were found to be similar to those in Model 1, which are both significantly negatively related to moderate- and high-street connectivity. In particular, we found the OR of depression onset in individuals who went out daily, walked for 30–59 min per day, walked for ≥ 60 min per day, seeing friends more than once per month, and engaged in social participation more than once per month to be 0.67 (95% CI 0.50–0.90), 0.81 (0.72–0.91), 0.69 (0.61–0.77), 0.76 (0.68–0.84), and 0.69 (0.63–0.76) times, respectively, less prone to depression onset than those did not.

**Table 2:** Logistic regression results for associations between depression and explanatory variables (Model 1^a^, Model 2^b^, and Model 3^c^; for full tables see Supplementary Tables 2-1-1, 2-1-2, 2-1-3, 2-2-1, 2-2-2, 2-2-3).

Characteristics	Model 1^a^	Model 2^b^	Model 3^c^
	OR (95% CI)		
**Intersection density (intersections/km**^2^)			
Low (≤ 155)	Ref.	Ref.	Ref.
Moderate (156–216)	0.97 (0.86–1.09)	0.97 (0.86–1.10)	0.99 (0.87–1.11)
High (≥ 217)	**0.83 (0.72–0.96)**	**0.82 (0.71–0.95)**	**0.83 (0.72–0.96)**
**Space syntax connectivity (numbers)**			
Low (< 2.7)	Ref.	Ref.	Ref.
Moderate (2.7–3.0)	**0.85 (0.76–0.95)**	**0.85 (0.76–0.96)**	**0.85 (0.76–0.96)**
High (≥ 3.0)	**0.86 (0.75–0.97)**	**0.85 (0.75–0.97)**	**0.84 (0.74–0.96)**

*Ref.* Reference group, *OR* Odds ratio, *95% CI* 95% Confidence interval.

*p*-Value = significant *p* values (< 0.05) are in bold.

^a^Model 1 controlled for sex, age, education, equivalent household income, marital status, living situation, driving status, land value, and population density.

^b^Model 2 controlled for sex, age, education, equivalent household income, marital status, living situation, driving status, land value, population density, frequency of going out, and duration of walking time.

^c^Model 3 controlled for sex, age, education, equivalent household income, marital status, living situation, driving status, land value, population density, frequency of seeing friends, and social participation.

The results for street connectivity and depression stratified by sex, population density, and latitude demonstrated that the point estimates of depression remain suppressed in the same direction. However, these results were partly significant for intersection density: male, residents in areas between 40° and 45° north, and areas with a high population density areas; for space syntax connectivity: both male and female, residents in areas between 30° and 35° N and between 40° and 45° N, areas with high and low-population density (Supplementary Tables 3-1, 3-2, 4-1, 4-2, 5-1, 5-2). We found that the interaction of street connectivity (intersection density and space syntax connectivity) with sex, population density, or latitude is nonsignificant (data not shown).

## Discussion

This longitudinal study evaluated associations between street connectivity (intersection density and space syntax connectivity) and the onset of depression during a 3-year follow-up study of older Japanese adults. Intersection density and space syntax connectivity were calculated differently, but they both represent connectivity. Our findings revealed an association between higher street connectivity and lower odds of depression onset. The results may have implications for urban design and highlight possible unintended benefits of intervention using built environments for depression risk among older adults. Given that poor mental health can lead to physical health diseases related to suicide and enormous economic burdens, the present result points to protective factors for approaches for preventing mental disorders^34^. Physical activities and social interaction were significantly negatively related to depression; however, after controlling for both factors, the relationship between street connectivity and depression onset remained. A plausible mechanism of this association operates through three facets, namely, physical activity, experiences of pedestrian movement, and sidewalks and cognitive function.

In a previous research, high street connectivity was associated with increased physical activity. For example, an Australian study reported that increases in street connectivity were associated with increased time spent walking among middle-aged residents^23^. Koohsari et al. (2017) mentioned that areas with better street connectivity are beneficial for walking for transport, partially because more commercial destinations are available and accessible^35^. Increased accessibility was associated with low depression and less stressful environments for aging^36^. Moreover, previous research found that older people living in areas with better access to health clinics and services were less likely to report depressive symptoms^31,37^. Our results indicate that frequent going out and walking exert a protective effect against depression. However, the association between street connectivity and depression onset remained significant after regression analyses was conducted by adding physical activities sequentially. We deemed that the factors of physical activities used are likely prone to bias from measurement, which is an aspect that we were unable to capture. For example, the measure for walking time is self-reported instead of objectively measured data, such as accelerometer assessment. In addition, the following responses to the questions on walking time are as follows: “less than 30 min,” “30–59 min,” “60–89 min,” and “90 min or more.” Thus, we deemed that the potential source of bias appeared in the measurement, whereas the categories of variables that may influence the relationship (street connectivity and depression) remained after adjusting for physical activities.

The association between street connectivity and depression remained after adjustment for the factors of physical activities and social interaction, which suggests that additional unobserved factors may explain this relationship, such as greenness, public transportation, and other experiences of pedestrian movement. Leslie and Cerin (2008) reported that perceived street connectivity is positively associated with social networks, including friends and neighbors^24^. Furthermore, in Japan, the findings of longitudinal studies indicated that frequent meetings with friends could prevent depression in a population of older adults^28^. In another Japanese study, community-level civic participation was inversely associated with the onset of depressive symptoms among older people^38^. Although the current study found that frequently seeing friends and attending activity groups is important and related to mental health, we were unable to establish that social interaction is a possible explanation for our findings. As such, the relationship between street connectivity and social interaction among older adults and the association between this relationship and depression requires further investigation. For other possibilities of the underlying mechanism between street connectivity and depression, we considered the experiences of pedestrian movement, such as traffic volume, seating facilities, access to public transportation, and greenness at the eye level of pedestrians^32,39–42^. According to a previous study in Hong Kong, high street connectivity was associated with increased depressive symptoms among older adults due to areas with heavy traffic volumes and less seating facilities^32^. Furthermore, James et al. (2017) reported that mental health could be negatively affected by high traffic density or poor quality of pedestrian infrastructure regardless of living in a neighborhood with a walkable environment^39^. Cerin et al. (2019) mentioned that well-connected street networks promote the availability of public transportation^40^, Yang et al. (2018) reported that more bus lines and stations effectively reduce depression among older American adults^41^. Other experiences of pedestrian movement, such as exposure to greenness, especially eye-level greenspace in tree-planting zones, may indicate a path between street connectivity and depression. Scholars proposed that eye-level greenspace quality plays an important role in mental health due to the benefit of greenspace, including capacity-restoring effect and stimulus on mental well-being. The findings of the China study suggest that the exposure of pedestrians to green space during walking is beneficial to mental health because the majority of daily street greenery can provide shade protection and offer a restorative environment^42^.

Recently, scholarly interest in the relationships between street network design and cognitive function has been increasing^26,43,44^, where one component of high street connectivity may be the increased number of sidewalks in street networks. In Japan, older adults living in urban areas with many sidewalks displayed low risks of dementia than those living in urban areas with few sidewalks^44^. Although street connectivity is concerned with the interconnectedness of segments; on the other hand, sidewalks denotes width, continuity, and obstacles^44^; in addition, simply adding sidewalks to the existing roads does not alter connectivity. However, high street connectivity and sidewalk environment support walking for transport; thus, we conducted a Pearson’s correlation coefficient analysis using street connectivity values and sidewalk area data from Tani et al.^44^. We identified a high correlation (*r* = 0.63, *p* < 0.001) between sidewalk areas and street connectivity.

In a 2-year longitudinal study, increased neighborhood connectivity was associated with high cognitive function in older adults^26^. Researchers highlighted that additional intersections denote less direct routes and more available ground area to cover. Thus, they suggested that the greater cognitive complexity required to navigate while walking strengthens the cognitive function of individuals who regularly walk in such areas^15,26^. Although previous research pointed to a bidirectional relationship between cognitive function and depression, a 36-month follow-up study reported that older people with dementia at baseline experienced a high prevalence and cumulative incidence of depressive symptoms than participants without mild Alzheimer's disease^45^. Meanwhile, a systematic review and meta-analysis of 29 longitudinal studies found that the associations between cognitive function and subsequent depression were significant^30^. Thus, we deemed that certain environmental factors related to cognitive function could also influence depression.

This study has several strengths. First, to our knowledge, this is the first longitudinal investigation of the relationship between street connectivity measured by intersection density and space syntax and depression among older adults. Second, the current sample size is larger than those in previous studies, as larger sample sizes provide more accurate mean values and a smaller margin of error. A systematic review focused on relationships between the neighborhood environment and depression among older people and reported that out of 73 articles, the sample size of 66% of the studies is less than 2500^9^. Third, we could use a large population-based longitudinal data set in which the effect of reverse causation was being controlled. Moreover, our data covered 17 municipalities from northern and southern Japan, encompassing both urban and rural areas, and thus, we were able to capture the full range of street connectivity across the areas we studied.

Nevertheless, this study has some limitations. First, space syntax calculations of street connectivity only count numbers of connected streets, so we could not consider individual area characteristics, such as the width, safety, or even existence of sidewalks. However, correlation analysis did show positive associations between street connectivity and sidewalk area. Second, we should have included climate factors such as snowfall and amount of rain as adjustment variables because the weather in the northern areas might have influenced the street construction, and climate factors might be associated with seasonal depression^46^. However, our sensitivity analysis of the findings stratified by latitude showed that the depression point estimate remained in the same direction of suppression. Third, the onset of depression between the baseline and the follow-up was based on responses to a self-administered questionnaire, which means that respondents could have experienced recall bias when answering or might not have answered truthfully because of social desirability bias^47^. Fourth, the loss of 60% of the participants should be noted because we excluded individuals without residential information and with GDS 5 and more at baseline to focus on the onset of depression. Fifth, street layout values were measured using an 800 m circular buffer, which may lead to bias. As Koohsari et al. (2021) noted, a circular buffer may not genuinely represent the attributes of the built environment to which participants were exposed^48^. Additionally, we cannot deny the possibility that certain participants would live outside the buffer, as the buffer is imposed by the administrative unit. However, 97% of administrative units are smaller than the 800 m circular buffer, and 3% of the administrative units have a roughly 50% probability that the participants reside inside the 800 m buffer. We could not access the actual addresses of the participants due to reasons of confidentiality; thus, future studies should replicate the findings across types and sizes of spatial buffers^47,48^. Sixth, this study classified the level of street connectivity based on distribution and classified it into three quartiles instead of using scientific criteria. The reason for this that previous research is insufficient to scientifically construct a cutoff for street connectivity. To test the appropriateness of the tertile, we analyzed the data per quintile, which was negatively significantly associated with depression onset but did not indicate a clear dose response (supplementary materials Tables 6-1, 6-2). The point estimates of depression remained suppressed in the same direction when examined as continuous values but were statistically nonsignificant. Notably, a previous study found a curvilinear association between intersection density and physical activity in an inverted U shape, thereby reporting that a high probability of walking behavior is associated with the threshold value of intersection density^49^. Greater street connectivity may not provide better mental health it may lead to increased concerns about traffic safety. We considered that this association does not necessarily appear, no matter what level of connectivity values we compare, but appears when we take the low-connectivity area as a reference and compare it with a higher one. Thus, a precise analysis of the cutoff for street connectivity is required in relation to depression.

Future researchers should focus on areas with low connectivity to further investigate the mechanisms that operate between street connectivity and other built environment elements regarding depression risk in older adults, including more measures related to street layout and neighborhood-level covariates. We established with this study that older adults in Japan who lived in areas with high street connectivity had a lower risk of depression onset during a 3-year study period from 2013 to 2016. We found that the association between street connectivity and depression held after adjustment for the factors of physical activities and social interaction, which suggests that additional unobserved factors may explain this relationship. With a high population of older adults in Japan, understanding how street layouts can influence mental health will be essential for promoting their long-term well-being and supporting the development of urban design. The clear connections between street connectivity and good mental health make this a valuable area for exploration.

## Methods

### Study participants

**Figure 1:**
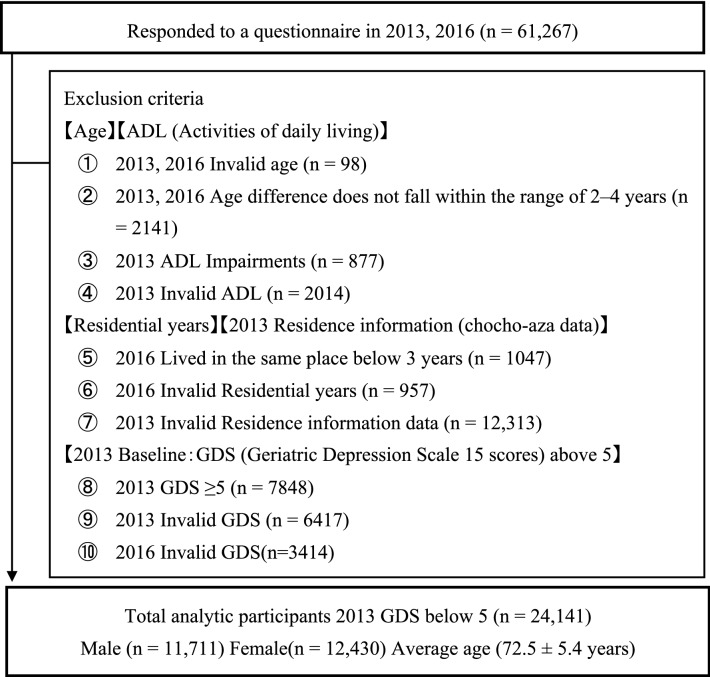
Flow of participants in the study.

We set the baseline in 2013 to perform a 3-year follow-up longitudinal study using panel data from the Japan Gerontological Evaluation Study (JAGES). The JAGES is a population-based epidemiological research initiative conducted on older people every 3 years since 2010. It focuses on the social determinants of health and the social/built environment^50^. The self-administered questionnaires were mailed to independent individuals aged ≥ 65 years across 24 municipalities in Japan in 2013 and 2016. The 2013 baseline JAGES was conducted between October and December 2013 with a total of 173,643 questionnaires distributed to older people and 126,781 returned and completed (response rate: 73.0%). The follow-up survey was conducted between October and December 2016 with a total of 154,496 questionnaires distributed, where 109,672 were returned and completed (response rate: 71.0%). 61,267 respondents completed the questionnaire in both years with an average follow-up period of 1,106 ± 9.4 days. In selecting the respondents, we used three exclusion criteria. First, we excluded any respondents who required assistance with daily living activities, with missing data on age, and whose age difference between baseline and after 3 years did not fall within the range of 2–4 years. Second, we excluded respondents with missing data on residential years or residence information (Chocho-aza point data: the smallest administrative unit used for the 1995 population census of Japan, roughly comparable to a European parish or a block group in the United States)^51^. Respondents not living in the same place at both survey time points were excluded, because they lack a consistent exposure to the same neighborhood over the 3-year period. Third, we excluded respondents with missing scores from the 15-item Geriatric Depression Scale (short form; GDS-15) in 2013 and 2016 and those with baseline (2013) GDS-15 score above 5^52^. We excluded the last group, because the study focuses on the potential association between street connectivity and depression onset instead of effects on existing depression. After excluding the aforementioned groups, 24,141 data of older Japanese people residing in rural and urban areas across the country (Fig. 1) were used for analysis.

### Outcome variable

 The outcome variable was depression, which was assessed using GDS-15^53^; the GDS-15 score ranges from 0 to 15 points. A score of 5 or more indicates depression. GDS-15 was developed for self-administration using a simple yes/no format, and its validity and reliability have been confirmed among older Japanese populations^52^. We defined participants as having developed depression during the follow-up period if their GDS-15 score was 5 or over at the 2016 survey.

### Explanatory variable

**Figure 2:**
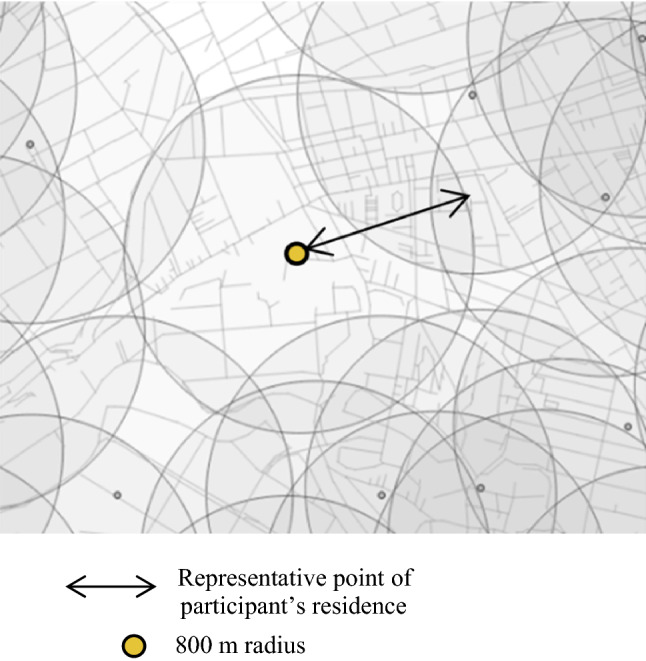
Chocho-aza point data and 800 m buffer.

**Figure 3:**
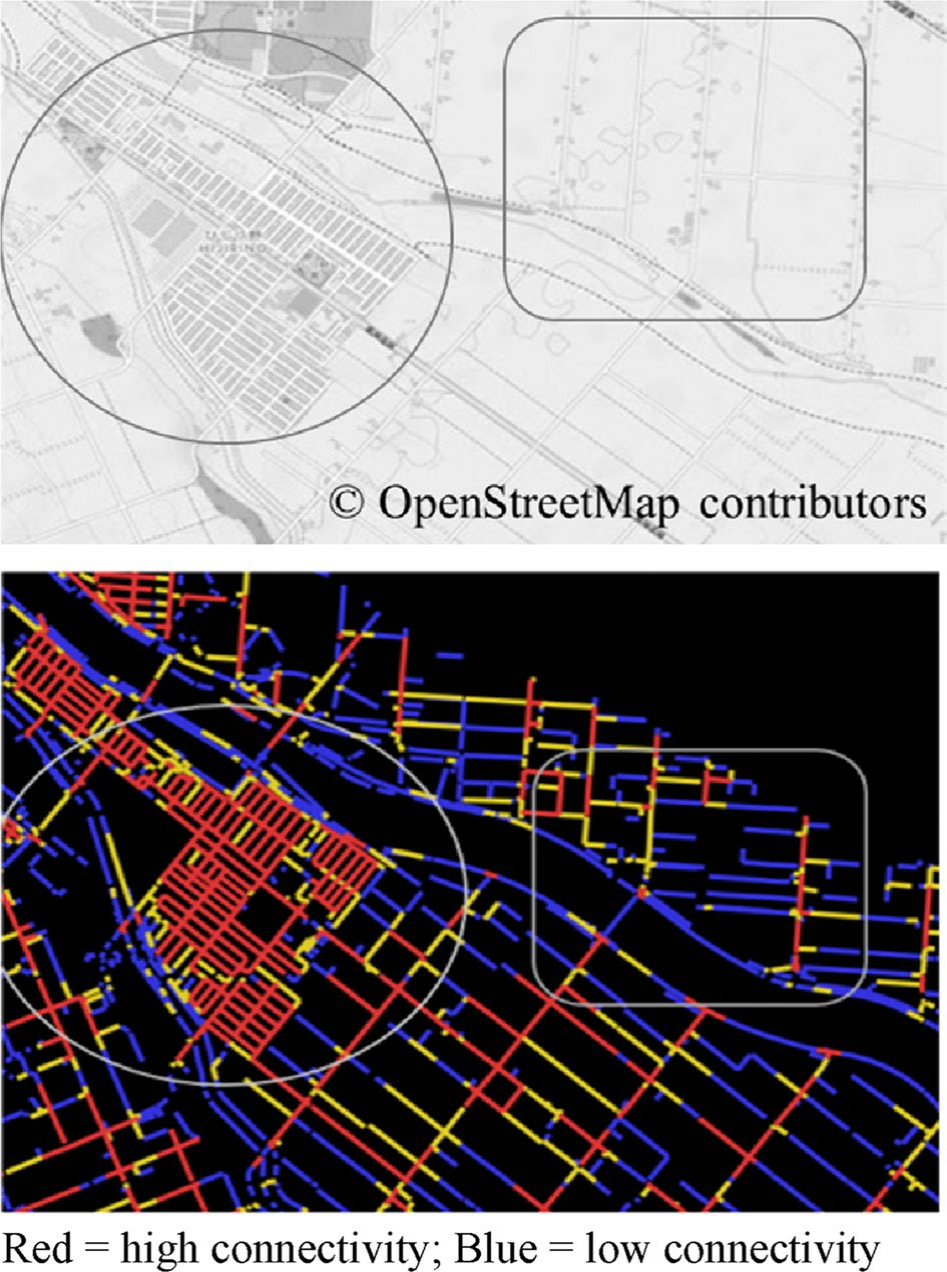
OpenStreetMap (top) and space syntax connectivity map (bottom).

**Figure 4:**
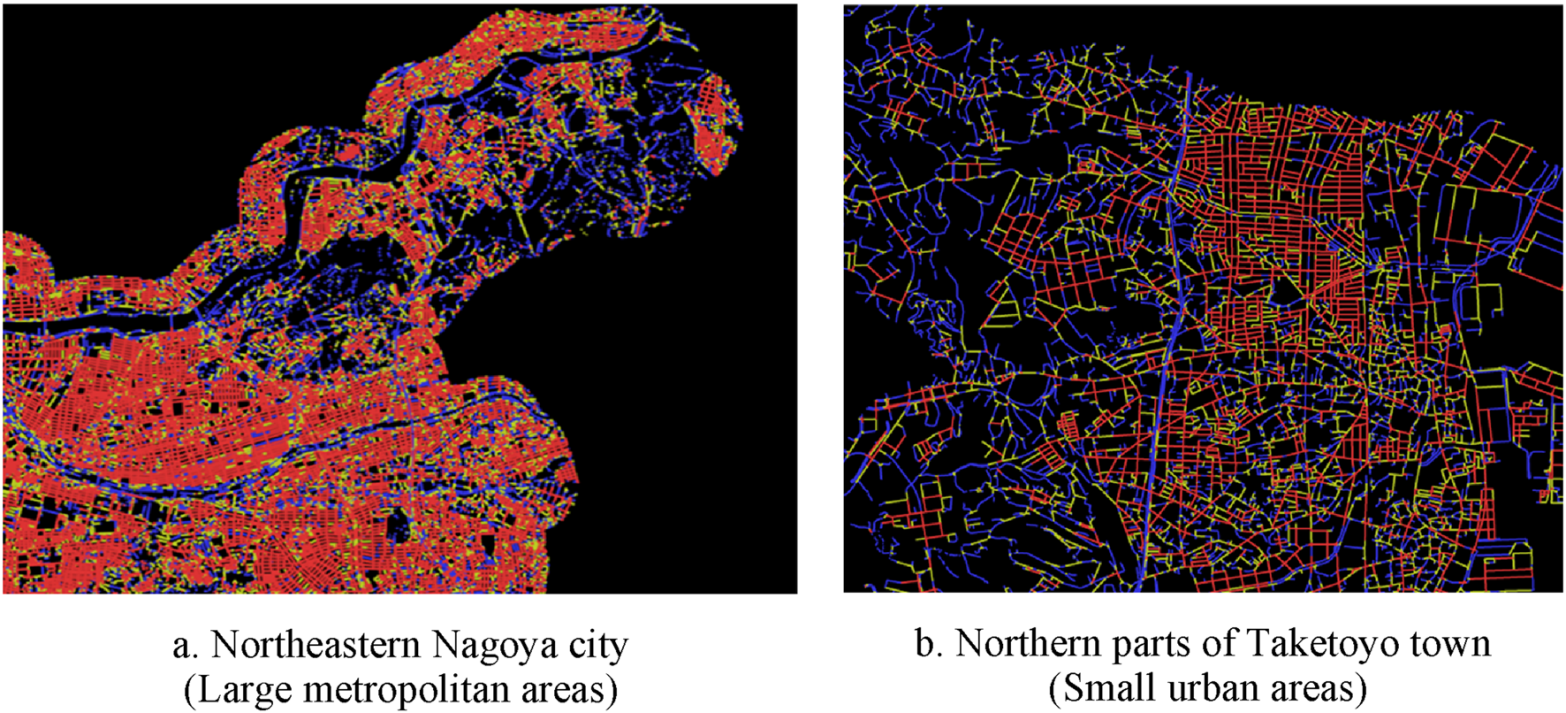
Space syntax connectivity maps of research municipalities. Panels (**a**) and (**b**) show areas of both low and high connectivity in both large metropolitan areas and small urban areas. High-population areas show higher street connectivity than low population areas.

Our explanatory variable was tertiles of street connectivity within 800 m of the center of each participant’s residence (representative point). We were unable to use the actual addresses of the participants. We used the latitude and longitude of a representative point (chocho-aza point data) in the participant’s neighborhood, which were identified through geocoding to the smallest administrative unit^51^. We set 800 m as the buffer to be consistent with previous research findings on the correlation between the behavior of older people and environmental outcomes^43,54^. Regarding street connectivity, two objective variables included: intersection density and space syntax connectivity (Both using 2013 street network data from Zenrin Co., Ltd. and simplified the data in ArcGIS ver. 10.3.1 (Environmental Systems Research Institute, ESRI, Redlands, CA, USA)). Intersection density was commonly used in previous studies, we computed it as the number of intersections (three-way or more) per network buffer 800 m circular buffer^25,32,33^. Space syntax connectivity measures the number of street segments that immediately directly connect to a street segment^55^. We used DepthMapX software (settings = Tulip Bin: 1024, Radius type: Metric) (University College London, n.d.), where the connectivity for each street segment was calculated using space syntax theory^56,57^, a method used to analyze urban space and developed areas at the University of London in the 1970s^58^. This method focuses on the street elements of connectivity and relationships and analyzes them mathematically using graphs^59,60^. Koohsari et al. opine that intersection density is a typical measure, and space syntax provides an alternative way to measure street connectivity. In active research predicting pedestrian movement (such as in a natural movement)^61^ and public health has used space syntax to investigate the relationships between street layout and health problems^26,43,62^. However, we found no previous study that has used it with depression. Third, we assigned street connectivity values to each street segment using QGIS ver. 3.8^63,64^ to calculate the mean street connectivity within 800 m of the center of the representative point of each participant’s residence (Fig. 2). The top panel of Fig. 3 displays a street map of a part of one target area, whereas the bottom panel presents a DepthMapX street connectivity diagram of the same area. In the diagram, red and blue indicate high and low street connectivity, respectively. Figure 4a,b present areas with low and high connectivity in both large metropolitan and small urban areas.

### Covariates

We controlled for demographics, socioeconomics, physical activity, social interaction, and regional characteristics which may likely correlate with depression. Demographic and socioeconomic covariates included sex (male or female), age (65–69, 70–74, 75–79, 80–84, or ≥ 85 years), equivalent household income (< 2, 2–3.99, or ≥ 4 million yen per year or missing), educational attainment (≤ 9 or ≥ 10 years or missing), marital status (married, widowed, divorced, never married, or missing), living status (living alone, living with others, or missing), driving status (car user, including receiving rides from others or non-car-user) and years of residence (< 10, 10–19, 20–29, 30–39, 40–49, and ≥ 50 years, or missing). We included the factors of physical activity and social interaction such as frequency of going out (annually, none, weekly, daily, or missing), duration of daily walking (< 30, 30–59, ≥ 60 min, or missing), frequency of seeing friends (more than once per month, less than once per month, or missing), and social participation (more than once per month, less than once per month, or missing). Social participation refers to individuals who participated in any of the 14 organized and regular activities (groups or clubs of volunteer, sports, leisure activity, senior citizen, neighborhood or resident association, study or cultural group, long-term care prevention or health-building activities, local events; activities that teach skills or pass on experiences to others, support older people requiring protection, long-term care, parents raising children, improvement in local living arrangements, or another group/organization) at least once per month.

The regional factors we measured were population density by quintile (< 3322 people/km^2^ school district, 3322–4528, 4528–9213, or > 9213) and land value by quintile (< 35,500 yen/m^2^, 35,500–65,100, 65,100–144,000, or > 144,000).The former was adjusted for urbanity, whereas the latter was controlled for neighborhood socioeconomic status. All covariates were assessed using a questionnaire in the baseline survey^10,16^. We categorized the covariates with missing data as missing, and analysis included participants with missing data on the covariates.

### Statistical analysis

The methods we used to analyze the study data included descriptive analysis and the chi-squared test to calculate the differences between depression and each variable. To identify the intermediate factors, we consider physical activities and social interaction as pathways between connectivity and depression. We tested three models using logistic regression analysis via forced entry and controlled for covariates in the model to examine the associations between street connectivity (intersection density and space syntax connectivity) and depression in older Japanese people. In Model 1, the explanatory variable, outcome variable, and covariates (sex, age, equivalent household income, educational attainment, marital status, living status, years of residence, driving status, population density, and land value) are included. Model 2 further adjusts the frequency of going out and duration of daily walking as variables of physical activities in Model 1. Finally, in Model 3, we added the frequency of seeing friends and social participation as social interaction variables to Model 1. We also used sensitivity analysis stratified by gender due to the fact that women suffer from depression more than males^65^. Moreover, stratification analyses of population density and latitude were conducted to evaluate the differences between areas and climates, where we deem that high-connectivity areas exist in areas with high population density or without heavy snow. Furthermore, research reported that the prevalence of depression is associated with seasonality, especially autumn and winter^46^. The interaction among sex, population density, latitude, and street connectivity are calculated. We calculated all statistics using Stata/MP 16.1 software (Stata Corp, College Station, TX, USA) with a significance level of 5%.

### Ethics approval and consent to participate

This study used data from the JAGES, which is a project conducted by the Nihon Fu-kushi University Center for Well-Being and Society. The JAGES project was approved by the Ethics Committee on Research of Human Subjects at Nihon Fukushi University (application number 10–05), and the JAGES 2013 survey has also been approved by the Ethics Committee at the Chiba University Faculty of Medicine (application number 13–14). Furthermore, JAGES 2016 was approved by the National Center for Geriatrics and Gerontology and Chiba University (receipt number 992, 2493). The JAGES project was approved by the Ethics Committee on Research of Human Subjects at Nihon Fukushi University (application number 10–05). The JAGES 2013 survey was approved by the Ethics Committee at the Chiba University Faculty of Medicine (application number 13–14), whereas the JAGES 2016 was approved by the National Center for Geriatrics and Gerontology and Chiba University (receipt number 992 and 2493). The participants were informed that participation was voluntary and that completing and returning the questionnaire via mail indicated consent to participate. We created an anonymized dataset. All methods were conducted according to relevant guidelines and regulations or according to the Declaration of Helsinki.

## Supplementary Information

Supplementary Information.

## Data Availability

The data that support the findings of this study are available from JAGES but restrictions apply to the availability of these data, which were used under license for the current study, and so are not publicly available. Data are however available from the authors upon reasonable request and with permission of JAGES. Please contact the data management committee of JAGES and email dataadmin@jages.net requests for access.
