# Supplementary material for: The association between street connectivity and depression among older Japanese adults: the JAGES longitudinal study
Source: Sci Rep. 2022 Aug 8;12:13533. doi: 10.1038/s41598-022-17650-w (PMC9360019; doi:10.1038/s41598-022-17650-w)
Supplement: Supplementary file 1 — Supplementary Information. [file 41598_2022_17650_MOESM1_ESM.docx]

**Supplementary**

**Table2-1-1:** Logistic Regression Results for the Associations between Depression and Explanatory Variables (Model 1^a^) (Exposure: Intersection density (intersections/km^2^–800m circular buffer))

**Table2-1-2:** Logistic Regression Results for the Associations between Depression and Explanatory Variables (Model 2^b^) (Exposure: Intersection density (intersections/km^2^–800m circular buffer))

**Table2-1-3:** Logistic Regression Results for the Associations between Depression and Explanatory Variables (Model 3^c^) (Exposure: Intersection density (intersections/km^2^–800m circular buffer))

**Table2-2-1:** Logistic Regression Results for the Associations between Depression and Explanatory Variables (Model 1^a^) (Exposure: Space syntax connectivity (numbers))

**Table2-2-2:** Logistic Regression Results for the Associations between Depression and Explanatory Variables (Model 2^b^) (Exposure: Space syntax connectivity (numbers))

**Table2-2-3:** Logistic Regression Results for the Associations between Depression and Explanatory Variables (Model 3^c^) (Exposure: Space syntax connectivity (numbers))

**Table 3-1:** Sensitivity Analysis Stratified by Latitude (Exposure: Intersection density)

**Table 4-1:** Sensitivity Analysis Stratified by Population Density (Exposure: Intersection density)

**Table 5-1:** Sensitivity Analysis Stratified by Gender (Exposure: Intersection density)

**Table 3-2:** Sensitivity Analysis Stratified by Latitude (Exposure: Space syntax connectivity)

**Table 4-2:** Sensitivity Analysis Stratified by Population Density (Exposure: Space syntax connectivity)  **Table 5-2:** Sensitivity Analysis Stratified by Gender (Exposure: Space syntax connectivity)

**Table 6-1:** Using Quintile and Continuous Value of Street Connectivity as Explanatory Variables

(Exposure: Intersection density)

**Table 6-2:** Using Quintile and Continuous Value of Street Connectivity as Explanatory Variables

(Exposure: Space syntax connectivity)

Table 2-1-1: Logistic Regression Results for the Associations between Depression and Explanatory Variables (Model 1 ^a^) (Exposure: Intersection density (intersections/km^2^–800m circular buffer))

| **Characteristics** | **OR** | **95% CI** | ***p*-Value** |
| --- | --- | --- | --- |
| Tertiles of intersection density (intersections/km^2^–800m circular buffer) | | | |
| Low (≤155) | Ref. |  |  |
| Moderate (156–216) | 0.97 | 0.86–1.09 | 0.616 |
| High (≥217) | 0.83 | 0.72–0.96 | 0.012 |
| Sex |  |  |  |
| Male | Ref. |  |  |
| Female | 0.95 | 0.86–1.04 | 0.259 |
| Age (years) |  |  |  |
| 65–69 | Ref. |  |  |
| 70–74 | 1.05 | 0.94–1.18 | 0.358 |
| 75–79 | 1.22 | 1.08–1.39 | 0.002 |
| 80–84 | 1.62 | 1.38–1.89 | <0.001 |
| ≥85 | 2.38 | 1.90–2.96 | <0.001 |
| Education (years) |  |  |  |
| ≤9 | Ref. |  |  |
| ≥10 | 0.81 | 0.73–0.89 | <0.001 |
| Missing | 0.72 | 0.45–1.13 | 0.155 |
| Equivalent household income (million yen) | | | |
| Low (<2.00) | Ref. |  |  |
| Middle (2.00–3.99) | 0.71 | 0.64–0.79 | <0.001 |
| High (≥4.00) | 0.53 | 0.45–0.63 | <0.001 |
| Missing | 0.9 | 0.78–1.04 | 0.157 |
| Marital status |  |  |  |
| Married | Ref. |  |  |
| Widowed | 0.98 | 0.85–1.13 | 0.746 |
| Divorced | 1.24 | 0.94–1.63 | 0.122 |
| Never married | 1.41 | 1.03–1.92 | 0.032 |
| Other/missing | 1.15 | 0.83–1.59 | 0.41 |

Table 2–1–1. Cont.

| **Characteristics** | **OR** | **95% CI** | ***p*-Value** |
| --- | --- | --- | --- |
| Living situation |  |  |  |
| Lives with others | Ref. |  |  |
| Lives alone | 0.98 | 0.82–1.17 | 0.837 |
| Missing | 0.92 | 0.74–1.15 | 0.465 |
| Driving status |  |  |  |
| Not a car user | Ref. |  |  |
| Car user | 0.81 | 0.72–0.91 | <0.001 |
| Missing | 1.18 | 0.50–2.82 | 0.701 |
| Years of residence |  |  |  |
| <10 | Ref. |  |  |
| 10–19 | 0.94 | 0.76–1.17 | 0.571 |
| 20–29 | 0.81 | 0.65–1.01 | 0.065 |
| 30–39 | 0.88 | 0.72–1.07 | 0.196 |
| 40–49 | 0.86 | 0.71–1.04 | 0.13 |
| ≥50 | 0.79 | 0.65–0.95 | 0.014 |
| Missing | 0.72 | 0.48–1.08 | 0.111 |
| Land value (yen/m^2^) |  |  |  |
| <35,500 | Ref. |  |  |
| 35,500–65,100 | 0.94 | 0.82–1.07 | 0.34 |
| 65,100–144,000 | 0.97 | 0.81–1.15 | 0.72 |
| >144,000 | 1.03 | 0.82–1.29 | 0.78 |
| Population density (people/km^2^) |  |  |  |
| <3322 | Ref. |  |  |
| 3322–4528 | 1.04 | 0.91–1.18 | 0.581 |
| 4528–9213 | 0.96 | 0.80–1.14 | 0.639 |
| >9213 | 0.98 | 0.79–1.23 | 0.885 |

Ref. = Reference group; OR = Odds ratio; 95% CI = 95% Confidence interval

^a^ Model 1 controlled for sex, age, education, equivalent household income, marital status, living situation, driving status, land value, and population density.

Table 2-1-2: Logistic Regression Results for the Associations between Depression and Explanatory Variables (Model 2 ^b^) (Exposure: Intersection density (intersections/km^2^–800m circular buffer))

| **Characteristics** | **OR** | **95% CI** | ***p*-Value** |
| --- | --- | --- | --- |
| Tertiles of intersection density (intersections/km^2^–800m circular buffer) | | | |
| Low (≤155) | Ref. |  |  |
| Moderate (156–216) | 0.97 | 0.86–1.10 | 0.645 |
| High (≥217) | 0.82 | 0.71–0.95 | 0.008 |
| Sex |  |  |  |
| Male | Ref. |  |  |
| Female | 0.94 | 0.85–1.03 | 0.191 |
| Age (years) |  |  |  |
| 65–69 | Ref. |  |  |
| 70–74 | 1.05 | 0.93–1.17 | 0.434 |
| 75–79 | 1.19 | 1.04–1.35 | 0.009 |
| 80–84 | 1.52 | 1.29–1.78 | <0.001 |
| ≥85 | 2.16 | 1.73–2.70 | <0.001 |
| Education (years) |  |  |  |
| ≤9 | Ref. |  |  |
| ≥10 | 0.81 | 0.73–0.89 | <0.001 |
| Missing | 0.69 | 0.43–1.10 | 0.116 |
| Equivalent household income (million yen) | | | |
| Low (<2.00) | Ref. |  |  |
| Middle (2.00–3.99) | 0.73 | 0.66–0.81 | <0.001 |
| High (≥4.00) | 0.55 | 0.47–0.65 | <0.001 |
| Missing | 0.91 | 0.79–1.05 | 0.209 |
| Marital status |  |  |  |
| Married | Ref. |  |  |
| Widowed | 0.97 | 0.84–1.12 | 0.708 |
| Divorced | 1.24 | 0.94–1.63 | 0.131 |
| Never married | 1.37 | 1.00–1.87 | 0.051 |
| Other/missing | 1.10 | 0.79–1.52 | 0.583 |
| Living situation |  |  |  |
| Lives with others | Ref. |  |  |
| Lives alone | 0.99 | 0.83–1.19 | 0.946 |
| Missing | 0.91 | 0.73–1.13 | 0.374 |

Table 2-1-2. Cont.

| Characteristics | OR | 95% CI | *p*-Value |
| --- | --- | --- | --- |
| Driving status |  |  |  |
| Not a car user | Ref. |  |  |
| Car user | 0.81 | 0.73–0.92 | 0.001 |
| Missing | 1.24 | 0.46–3.38 | 0.674 |
| Years of residence |  |  |  |
| <10 | Ref. |  |  |
| 10–19 | 0.94 | 0.76–1.17 | 0.570 |
| 20–29 | 0.81 | 0.65–1.01 | 0.065 |
| 30–39 | 0.88 | 0.72–1.07 | 0.201 |
| 40–49 | 0.87 | 0.72–1.06 | 0.158 |
| ≥50 | 0.80 | 0.67–0.97 | 0.024 |
| Missing | 0.72 | 0.48–1.09 | 0.117 |
| Land value (yen/m^2^) |  |  |  |
| <35,500 | Ref. |  |  |
| 35,500–65,100 | 0.94 | 0.82–1.07 | 0.353 |
| 65,100–144,000 | 0.99 | 0.83–1.18 | 0.890 |
| >144,000 | 1.05 | 0.84–1.32 | 0.676 |
| Population density (people/km^2^) |  |  |  |
| <3322 | Ref. |  |  |
| 3322–4528 | 1.03 | 0.90–1.17 | 0.706 |
| 4528–9213 | 0.94 | 0.79–1.12 | 0.475 |
| >9213 | 0.98 | 0.78–1.22 | 0.826 |
| Frequency of going out |  |  |  |
| Annually None | Ref. |  |  |
| Weekly | 0.83 | 0.62–1.13 | 0.245 |
| Daily | 0.66 | 0.49–0.89 | 0.006 |
| Missing | 0.97 | 0.52–1.81 | 0.918 |
| Duration of daily walking, min |  |  |  |
| Low (<30) | Ref. |  |  |
| Moderate (30–59) | 0.81 | 0.72–0.91 | 0.001 |
| High (≥60) | 0.69 | 0.61–0.77 | <0.001 |
| Missing | 0.55 | 0.32–0.96 | 0.037 |

Ref. = Reference group; OR = Odds ratio; 95% CI = 95% Confidence interval

^b^ Model 2 controlled for sex, age, education, equivalent household income, marital status, living situation, driving status, land value, population density, frequency of going out, and duration of walking time.

Table 2-1-3: Logistic Regression Results for the Associations between Depression and Explanatory Variables (Model 3 ^c^) (Exposure: Intersection density (intersections/km^2^–800m circular buffer))

| **Characteristics** | **OR** | **95% CI** | ***p*-Value** |
| --- | --- | --- | --- |
| Tertiles of intersection density (intersections/km^2^–800m circular buffer) | | | |
| Low (≤155) | Ref. |  |  |
| Moderate (156–216) | 0.99 | 0.87–1.11 | 0.812 |
| High (≥217) | 0.83 | 0.72–0.96 | 0.014 |
| Sex |  |  |  |
| Male | Ref. |  |  |
| Female | 1.02 | 0.92–1.12 | 0.716 |
| Age (years) |  |  |  |
| 65–69 | Ref. |  |  |
| 70–74 | 1.08 | 0.96–1.20 | 0.196 |
| 75–79 | 1.25 | 1.10–1.42 | 0.001 |
| 80–84 | 1.63 | 1.39–1.91 | <0.001 |
| ≥85 | 2.24 | 1.79–2.80 | <0.001 |
| Education (years) |  |  |  |
| ≤9 | Ref. |  |  |
| ≥10 | 0.85 | 0.77–0.93 | 0.001 |
| Missing | 0.72 | 0.45–1.15 | 0.171 |
| Equivalent household income (million yen) | |  |  |
| Low (<2.00) | Ref. |  |  |
| Middle (2.00–3.99) | 0.73 | 0.66–0.81 | <0.001 |
| High (≥4.00) | 0.55 | 0.46–0.64 | <0.001 |
| Missing | 0.90 | 0.78–1.04 | 0.151 |
| Marital status |  |  |  |
| Married | Ref. |  |  |
| Widowed | 0.98 | 0.85–1.13 | 0.792 |
| Divorced | 1.21 | 0.92–1.60 | 0.167 |
| Never married | 1.31 | 0.96–1.80 | 0.090 |
| Other/missing | 1.13 | 0.81–1.56 | 0.481 |
| Living situation |  |  |  |
| Lives with others | Ref. |  |  |
| Lives alone | 1.04 | 0.87–1.24 | 0.657 |
| Missing | 0.94 | 0.75–1.16 | 0.563 |

Table 2-1-3. Cont.

| Characteristics | OR | 95% CI | *p*-Value |
| --- | --- | --- | --- |
| Driving status |  |  |  |
| Not a car user | Ref. |  |  |
| Car user | 0.83 | 0.74–0.94 | 0.002 |
| Missing | 1.12 | 0.47–2.66 | 0.804 |
| Years of residence |  |  |  |
| <10 | Ref. |  |  |
| 10–19 | 0.94 | 0.75–1.16 | 0.571 |
| 20–29 | 0.83 | 0.67–1.04 | 0.106 |
| 30–39 | 0.90 | 0.74–1.10 | 0.324 |
| 40–49 | 0.90 | 0.74–1.08 | 0.266 |
| ≥50 | 0.83 | 0.68–1.00 | 0.053 |
| Missing | 0.74 | 0.49–1.11 | 0.143 |
| Land value (yen/m^2^) |  |  |  |
| <35,500 | Ref. |  |  |
| 35,500–65,100 | 0.93 | 0.82–1.06 | 0.327 |
| 65,100–144,000 | 0.97 | 0.81–1.15 | 0.719 |
| >144,000 | 1.04 | 0.83–1.30 | 0.718 |
| Population density (people/km^2^) |  |  |  |
| <3322 | Ref. |  |  |
| 3322–4528 | 1.05 | 0.92–1.19 | 0.484 |
| 4528–9213 | 0.96 | 0.81–1.15 | 0.68 |
| >9213 | 0.99 | 0.79–1.24 | 0.944 |
| Frequency of seeing friends |  |  |  |
| Less than once per month | Ref. |  |  |
| More than once per month | 0.76 | 0.68–0.84 | <0.001 |
| Missing | 0.88 | 0.68–1.14 | 0.335 |
| Social participation |  |  |  |
| Less than once per month | Ref. |  |  |
| More than once per month | 0.69 | 0.63–0.76 | <0.001 |
| Missing | 0.96 | 0.81–1.13 | 0.616 |

Ref. = Reference group; OR = Odds ratio; 95% CI = 95% Confidence interval

^c^ Model 3 controlled for sex, age, education, equivalent household income, marital status, living situation, driving status, land value, population density, frequency of seeing friends, and social participation.

Table 2-2-1: Logistic Regression Results for the Associations between Depression and Explanatory Variables (Model 1 ^a^) (Exposure: Space syntax connectivity (numbers))

| **Characteristics** | **OR** | **95% CI** | ***p*-Value** |
| --- | --- | --- | --- |
| Space syntax connectivity (numbers) |  |  |  |
| Low (<2.7) | Ref. |  |  |
| Moderate (2.7–3.0) | 0.85 | 0.76–0.95 | 0.005 |
| High (≥3.0) | 0.86 | 0.75–0.97 | 0.019 |
| Sex |  |  |  |
| Male | Ref. |  |  |
| Female | 0.95 | 0.86–1.04 | 0.260 |
| Age (years) |  |  |  |
| 65–69 | Ref. |  |  |
| 70–74 | 1.05 | 0.94–1.18 | 0.361 |
| 75–79 | 1.22 | 1.08–1.39 | 0.002 |
| 80–84 | 1.62 | 1.38–1.90 | <0.001 |
| ≥85 | 2.38 | 1.91–2.97 | <0.001 |
| Education (years) |  |  |  |
| ≤9 | Ref. |  |  |
| ≥10 | 0.81 | 0.73–0.89 | <0.001 |
| Missing | 0.71 | 0.45–1.13 | 0.152 |
| Equivalent household income (million yen) | | | |
| Low (<2.00) | Ref. |  |  |
| Middle (2.00–3.99) | 0.72 | 0.65–0.79 | <0.001 |
| High (≥4.00) | 0.54 | 0.46–0.64 | <0.001 |
| Missing | 0.91 | 0.79–1.04 | 0.175 |
| Marital status |  |  |  |
| Married | Ref. |  |  |
| Widowed | 0.98 | 0.85–1.13 | 0.760 |
| Divorced | 1.25 | 0.95–1.64 | 0.111 |
| Never married | 1.42 | 1.04–1.94 | 0.029 |
| Other/missing | 1.15 | 0.83–1.60 | 0.399 |

Table 2-2-1. Cont.

| **Characteristics** | **OR** | **95% CI** | ***p*-Value** |
| --- | --- | --- | --- |
| Living situation |  |  |  |
| Lives with others | Ref. |  |  |
| Lives alone | 0.98 | 0.82–1.17 | 0.792 |
| Missing | 0.92 | 0.74–1.14 | 0.431 |
| Driving status |  |  |  |
| Not a car user | Ref. |  |  |
| Car user | 0.81 | 0.72–0.91 | <0.001 |
| Missing | 1.18 | 0.49–2.80 | 0.714 |
| Years of residence |  |  |  |
| <10 | Ref. |  |  |
| 10–19 | 0.94 | 0.76–1.16 | 0.558 |
| 20–29 | 0.81 | 0.65–1.01 | 0.060 |
| 30–39 | 0.87 | 0.72–1.06 | 0.180 |
| 40–49 | 0.85 | 0.70–1.03 | 0.107 |
| ≥50 | 0.77 | 0.64–0.94 | 0.008 |
| Missing | 0.71 | 0.47–1.07 | 0.099 |
| Land value (yen/m^2^) |  |  |  |
| <35,500 | Ref. |  |  |
| 35,500–65,100 | 0.95 | 0.83–1.09 | 0.497 |
| 65,100–144,000 | 0.97 | 0.82–1.15 | 0.724 |
| >144,000 | 1.00 | 0.81–1.25 | 0.966 |
| Population density (people/km^2^) |  |  |  |
| <3322 | Ref. |  |  |
| 3322–4528 | 1.04 | 0.92–1.19 | 0.531 |
| 4528–9213 | 0.99 | 0.83–1.17 | 0.878 |
| >9213 | 0.97 | 0.77–1.21 | 0.778 |

Ref. = Reference group; OR = Odds ratio; 95% CI = 95% Confidence interval

^a^ Model 1 controlled for sex, age, education, equivalent household income, marital status, living situation, driving status, land value, and population density.

Table 2-2-2: Logistic Regression Results for the Associations between Depression and Explanatory Variables (Model 2 ^b^) (Exposure: Space syntax connectivity (numbers))

| **Characteristics** | **OR** | **95% CI** | ***p*-Value** |
| --- | --- | --- | --- |
| Space syntax connectivity (numbers) |  |  |  |
| Low (<2.7) | Ref. |  |  |
| Moderate (2.7–3.0) | 0.85 | 0.76–0.96 | 0.007 |
| High (≥3.0) | 0.85 | 0.75–0.97 | 0.016 |
| Sex |  |  |  |
| Male | Ref. |  |  |
| Female | 0.94 | 0.85–1.03 | 0.192 |
| Age (years) |  |  |  |
| 65–69 | Ref. |  |  |
| 70–74 | 1.05 | 0.93–1.17 | 0.44 |
| 75–79 | 1.19 | 1.04–1.35 | 0.01 |
| 80–84 | 1.52 | 1.30–1.78 | <0.001 |
| ≥85 | 2.16 | 1.73–2.70 | <0.001 |
| Education (years) |  |  |  |
| ≤9 | Ref. |  |  |
| ≥10 | 0.81 | 0.73–0.89 | <0.001 |
| Missing | 0.69 | 0.43–1.10 | 0.116 |
| Equivalent household income (million yen) | | | |
| Low (<2.00) | Ref. |  |  |
| Middle (2.00–3.99) | 0.73 | 0.66–0.81 | <0.001 |
| High (≥4.00) | 0.55 | 0.47–0.66 | <0.001 |
| Missing | 0.92 | 0.80–1.06 | 0.232 |
| Marital status |  |  |  |
| Married | Ref. |  |  |
| Widowed | 0.97 | 0.85–1.12 | 0.728 |
| Divorced | 1.24 | 0.95–1.64 | 0.119 |
| Never married | 1.37 | 1.00–1.88 | 0.047 |
| Other/missing | 1.1 | 0.79–1.53 | 0.563 |
| Living situation |  |  |  |
| Lives with others | Ref. |  |  |
| Lives alone | 0.99 | 0.83–1.18 | 0.901 |
| Missing | 0.90 | 0.72–1.12 | 0.343 |

Table 2-2-2. Cont.

| Characteristics | OR | 95% CI | *p*-Value |
| --- | --- | --- | --- |
| Driving status |  |  |  |
| Not a car user | Ref. |  |  |
| Car user | 0.82 | 0.73–0.92 | 0.001 |
| Missing | 1.22 | 0.45–3.33 | 0.694 |
| Years of residence |  |  |  |
| <10 | Ref. |  |  |
| 10–19 | 0.94 | 0.76–1.16 | 0.555 |
| 20–29 | 0.81 | 0.65–1.01 | 0.060 |
| 30–39 | 0.87 | 0.72–1.07 | 0.184 |
| 40–49 | 0.86 | 0.71–1.04 | 0.130 |
| ≥50 | 0.79 | 0.65–0.95 | 0.014 |
| Missing | 0.71 | 0.47–1.07 | 0.104 |
| Land value (yen/m^2^) |  |  |  |
| <35,500 | Ref. |  |  |
| 35,500–65,100 | 0.95 | 0.83–1.09 | 0.473 |
| 65,100–144,000 | 0.98 | 0.83–1.17 | 0.862 |
| >144,000 | 1.02 | 0.81–1.27 | 0.888 |
| Population density (people/km^2^) |  |  |  |
| <3322 | Ref. |  |  |
| 3322–4528 | 1.03 | 0.91–1.17 | 0.644 |
| 4528–9213 | 0.97 | 0.81–1.15 | 0.706 |
| >9213 | 0.96 | 0.77–1.20 | 0.724 |
| Frequency of going out |  |  |  |
| Annually None | Ref. |  |  |
| Weekly | 0.84 | 0.62–1.14 | 0.273 |
| Daily | 0.67 | 0.50–0.90 | 0.008 |
| Missing | 0.99 | 0.52–1.85 | 0.963 |
| Duration of daily walking, min |  |  |  |
| Low (<30) | Ref. |  |  |
| Moderate (30–59) | 0.81 | 0.72–0.91 | 0.001 |
| High (≥60) | 0.69 | 0.61–0.77 | <0.001 |
| Missing | 0.55 | 0.32–0.97 | 0.038 |

Ref. = Reference group; OR = Odds ratio; 95% CI = 95% Confidence interval

^b^ Model 2 controlled for sex, age, education, equivalent household income, marital status, living situation, driving status, land value, population density, frequency of going out, and duration of walking time.

Table 2-2-3: Logistic Regression Results for the Associations between Depression and Explanatory Variables (Model 3 ^c^) (Exposure: Space syntax connectivity (numbers))

| **Characteristics** | **OR** | **95% CI** | ***p*-Value** |
| --- | --- | --- | --- |
| Space syntax connectivity (numbers) |  |  |  |
| Low (<2.7) | Ref. |  |  |
| Moderate (2.7–3.0) | 0.85 | 0.76–0.96 | 0.007 |
| High (≥3.0) | 0.84 | 0.74–0.96 | 0.011 |
| Sex |  |  |  |
| Male | Ref. |  |  |
| Female | 1.02 | 0.92–1.12 | 0.717 |
| Age (years) |  |  |  |
| 65–69 | Ref. |  |  |
| 70–74 | 1.08 | 0.96–1.20 | 0.199 |
| 75–79 | 1.25 | 1.10–1.42 | 0.001 |
| 80–84 | 1.63 | 1.39–1.91 | <0.001 |
| ≥85 | 2.24 | 1.79–2.80 | <0.001 |
| Education (years) |  |  |  |
| ≤9 | Ref. |  |  |
| ≥10 | 0.85 | 0.77–0.93 | 0.001 |
| Missing | 0.72 | 0.45–1.15 | 0.169 |
| Equivalent household income (million yen) | |  |  |
| Low (<2.00) | Ref. |  |  |
| Middle (2.00–3.99) | 0.73 | 0.66–0.81 | <0.001 |
| High (≥4.00) | 0.55 | 0.47–0.65 | <0.001 |
| Missing | 0.91 | 0.79–1.04 | 0.169 |
| Marital status |  |  |  |
| Married | Ref. |  |  |
| Widowed | 0.98 | 0.85–1.13 | 0.813 |
| Divorced | 1.22 | 0.93–1.61 | 0.151 |
| Never married | 1.32 | 0.96–1.80 | 0.084 |
| Other/missing | 1.13 | 0.81–1.57 | 0.471 |
| Living situation |  |  |  |
| Lives with others | Ref. |  |  |
| Lives alone | 1.04 | 0.87–1.24 | 0.689 |
| Missing | 0.93 | 0.75–1.16 | 0.524 |

Table 2-2-3. Cont.

| Characteristics | OR | 95% CI | *p*-Value |
| --- | --- | --- | --- |
| Driving status |  |  |  |
| Not a car user | Ref. |  |  |
| Car user | 0.84 | 0.75–0.94 | 0.003 |
| Missing | 1.11 | 0.47–2.65 | 0.811 |
| Years of residence |  |  |  |
| <10 | Ref. |  |  |
| 10–19 | 0.94 | 0.76–1.16 | 0.562 |
| 20–29 | 0.83 | 0.67–1.04 | 0.100 |
| 30–39 | 0.90 | 0.74–1.10 | 0.304 |
| 40–49 | 0.89 | 0.73–1.08 | 0.229 |
| ≥50 | 0.81 | 0.67–0.98 | 0.034 |
| Missing | 0.73 | 0.49–1.10 | 0.130 |
| Land value (yen/m^2^) |  |  |  |
| <35,500 | Ref. |  |  |
| 35,500–65,100 | 0.95 | 0.83–1.09 | 0.476 |
| 65,100–144,000 | 0.97 | 0.82–1.16 | 0.761 |
| >144,000 | 1.02 | 0.81–1.27 | 0.894 |
| Population density (people/km^2^) |  |  |  |
| <3322 | Ref. |  |  |
| 3322–4528 | 1.06 | 0.93–1.20 | 0.408 |
| 4528–9213 | 1.00 | 0.84–1.19 | 0.999 |
| >9213 | 0.99 | 0.79–1.23 | 0.897 |
| Frequency of seeing friends |  |  |  |
| Less than once per month | Ref. |  |  |
| More than once per month | 0.76 | 0.68–0.84 | <0.001 |
| Missing | 0.88 | 0.68–1.14 | 0.334 |
| Social participation |  |  |  |
| Less than once per month | Ref. |  |  |
| More than once per month | 0.69 | 0.63–0.76 | <0.001 |
| Missing | 0.96 | 0.81–1.14 | 0.669 |

Ref. = Reference group; OR = Odds ratio; 95% CI = 95% Confidence interval

^c^ Model 3 controlled for sex, age, education, equivalent household income, marital status, living situation, driving status, land value, population density, frequency of seeing friends, and social participation.

Table 3-1: Sensitivity Analysis Stratified by Latitude (Exposure: Intersection density)

| Latitude ^1)^  Intersection density | Model 1 | Model 2 | Model 3 |
| --- | --- | --- | --- |
|  | OR(95% CI) | OR(95% CI) | OR(95% CI) |
| Between 40° and 45° north (n = 2,269) | | | |
| Low (≤154) | Ref. | | |
| Moderate (155–211) | 0.83(0.57–1.19) | 0.80(0.55–1.16) | 0.85(0.59–1.23) |
| High (≥216) | **0.41(0.19–0.91)** | **0.41(0.17–0.92)** | **0.44(0.20–0.98)** |
| Between 35°and 40° north (n = 14,194) | | | |
| Low (≤154) | Ref. | | |
| Moderate (155–216) | 1.09(0.89–1.33) | 1.08(0.89–1.32) | 1.10(0.90–1.34) |
| High (≥217) | 0.88(0.72–1.08) | 0.87(0.71–1.06) | 0.88(0.72–1.07) |
| Between 30°and 35° north (n = 7,678) | | | |
| Low (≤155) | Ref. | | |
| Moderate (156–216) | 0.88 (0.73–1.08) | 0.90(0.74–1.09) | 0.90(0.74–1.10) |
| High (≥217) | 1.12 (0.77–1.62) | 1.12(0.77–1.62) | 1.14(0.79–1.67) |
| Table 4-1: Sensitivity Analysis Stratified by Population Density  (Exposure: Intersection density) | | | |
| Population density ^2)^  Intersection density | Model 1 | Model 2 | Model 3 |
|  | OR(95% CI) | OR(95% CI) | OR(95% CI) |
| High population density areas(n = 11,642) | | | |
| Low (≤202) | Ref. | | |
| Moderate (203–241) | 0.93(0.79–1.09) | 0.92(0.78–1.08) | 0.91(0.77–1.08) |
| High (≥242) | **0.79(0.66–0.94)** | **0.78(0.65–0.93)** | **0.76(0.63–0.91)** |
| Low population density areas (n = 12,498) | | | |
| Low (≤109) | Ref. | | |
| Moderate (110–172) | 0.92(0.78–1.06) | 0.92(0.79–1.07) | 0.92(0.79–1.06) |
| High (≥173) | 0.87(0.74–1.04) | 0.87(0.73–1.03) | 0.88(0.74–1.05) |
| Table 5-1: Sensitivity Analysis Stratified by Gender (Exposure: Intersection density) | | | |
| Gender  Intersection density | Model 1 | Model 2 | Model 3 |
|  | OR(95% CI) | OR(95% CI) | OR(95% CI) |
| Male (n = 11,711) | | | |
| Low (≤155) | Ref. | | |
| Moderate (156–216) | 0.94(0.79–1.12) | 0.94(0.79–1.12) | 0.95(0.79–1.13) |
| High (≥217) | **0.74(0.59–0.91)** | **0.73(0.59–0.89)** | **0.73(0.59–0.90)** |
| Female (n = 12,430) | | | |
| Low (≤155) | Ref. | | |
| Moderate (156–216) | 0.99(0.84–1.18) | 0.99(0.84–1.18) | 1.02(0.86–1.21) |
| High (≥217) | 0.92(0.75–1.12) | 0.91(0.75–1.11) | 0.93(0.76–1.14) |

Ref. = Reference group; OR = Odds ratio; 95% CI = 95% Confidence interval;
*p*-Value = significant p-values (<0.05) are in bold

1) Logistic regression ﻿analysis via forced entry adjusting for sex, age, equivalent household income, educational attainment, marital status, living status, driving status, frequency of going out, land value, population density, and duration of walking.

2) Stratified by population density in median: ﻿ <4528 or >4528 people/ km^2^ school district.

Logistic regression ﻿analysis via forced entry adjusting for sex, age, equivalent household income, educational attainment, marital status, living status, driving status, frequency of going out, land value, and duration of walking.

Table 3. Sensitivity analysis stratified by latitude

Model 1 controlled for sex, age, education, equivalent household income, marital status, living situation, driving status, land value, and population density.

Model 2 controlled for sex, age, education, equivalent household income, marital status, living situation, driving status, land value, population density, frequency of going out, and duration of walking time.

Model 3 controlled for sex, age, education, equivalent household income, marital status, living situation, driving status, land value, population density, frequency of seeing friends, and social participation.

Table 4. Sensitivity analysis stratified by population density.

Model 1 controlled for sex, age, education, equivalent household income, marital status, living situation, driving status, and land value.

Model 2 controlled for sex, age, education, equivalent household income, marital status, living situation, driving status, land value, frequency of going out, and duration of walking time.

Model 3 controlled for sex, age, education, equivalent household income, marital status, living situation, driving status, land value, frequency of seeing friends, and social participation.

* We adjusted the connectivity criteria in urban and rural areas based on population density.

Table 5. Sensitivity analysis stratified by gender

Model 1 controlled for age, education, equivalent household income, marital status, living situation, driving status, and land value.

Model 2 controlled for age, education, equivalent household income, marital status, living situation, driving status, land value, frequency of going out, and duration of walking time.

Model 3 controlled for age, education, equivalent household income, marital status, living situation, driving status, land value, frequency of seeing friends, and social participation.

Table 3-2: Sensitivity Analysis Stratified by Latitude (Exposure: Space syntax connectivity)

| Latitude ^1)^  Space syntax connectivity | Model 1 | Model 2 | Model 3 |
| --- | --- | --- | --- |
|  | OR(95% CI) | OR(95% CI) | OR(95% CI) |
| Between 40° and 45° north (n = 2,269) | | | |
| Low (<2.7) | Ref. | | |
| Moderate (2.7–3.0) | **0.69(0.43–0.99)** | **0.68(0.47–0.97)** | 0.72(0.50–1.04) |
| High (>3.0) | 0.95(0.63–1.42) | 0.91(0.60–1.37) | 0.97(0.65–1.47) |
| Between 35°and 40° north (n = 14,194) | | | |
| Low (<2.7) | Ref. | | |
| Moderate (2.7–3.0) | 0.95(0.80–1.12) | 0.95(0.80–1.12) | 0.95(0.80–1.12) |
| High (>3.0) | 0.94(0.78–1.12) | 0.92(0.77–1.10) | 0.92(0.77–1.11) |
| Between 30°and 35° north (n = 7,678) | | | |
| Low (<2.7) | Ref. | | |
| Moderate (2.7–3.0) | **0.77(0.61–0.97)** | **0.79(0.63–0.99)** | **0.79(0.63–0.99)** |
| High (>3.0) | **0.67(0.50–0.93)** | **0.69(0.52–0.93)** | **0.67(0.50–0.90)** |
| Table 4-2: Sensitivity Analysis Stratified by Population Density  (Exposure: Space syntax connectivity) | | | |
| Population density ^2)^  Space syntax connectivity | Model 1 | Model 2 | Model 3 |
|  | OR(95% CI) | OR(95% CI) | OR(95% CI) |
| High population density areas (n = 11,642) | | | |
| Low (<2.88) | Ref. | | |
| Moderate (2.88–3.34) | **0.79(0.66**–**0.95)** | **0.79(0.66**–**0.95)** | **0.78(0.65**–**0.94)** |
| High (>3.34) | 1.00(0.86–1.21) | 1.00(0.85–1.20) | 0.98(0.83–1.17) |
| Low population density areas (n = 12,498) | | | |
| Low (<2.48) | Ref. | | |
| Moderate (2.48–2.83) | 0.88(0.76–1.03) | 0.88(0.76–1.03) | 0.89(0.76–1.04) |
| High (>2.83) | **0.83(0.71**–**0.97)** | **0.83(0.71**–**0.97)** | **0.83(0.71**–**0.98)** |
| Table 5-2: Sensitivity Analysis Stratified by Gender (Exposure: Space syntax connectivity) | | | |
| Gender  Space syntax connectivity | Model 1 | Model 2 | Model 3 |
|  | OR(95% CI) | OR(95% CI) | OR(95% CI) |
| Male (n = 11,711) | | | |
| Low (<2.7) | Ref. | | |
| Moderate (2.7–3.0) | 0.87(0.73–1.02) | 0.87(0.74–1.04) | 0.87(0.74–1.03) |
| High (>3.0) | 0.84(0.69–1.01) | **0.83(0.68–1.00)** | **0.82(0.68–0.99)** |
| Female (n = 12,430) | | | |
| Low (<2.7) | Ref. | | |
| Moderate (2.7–3.0) | **0.83(0.71**–**0.97)** | **0.83(0.71**–**0.98)** | **0.83(0.56**–**0.74)** |
| High (>3.0) | 0.86(0.72–1.03) | 0.86(0.72–1.03) | 0.86(0.68–1.09) |

Ref. = Reference group; OR = Odds ratio; 95% CI = 95% Confidence interval;
*p*-Value = significant p-values (<0.05) are in bold

1) Logistic regression ﻿analysis via forced entry adjusting for sex, age, equivalent household income, educational attainment, marital status, living status, driving status, frequency of going out, land value, population density, and duration of walking.

2) Stratified by population density in median: ﻿ <4528 or >4528 people/ km^2^ school district.

Logistic regression ﻿analysis via forced entry adjusting for sex, age, equivalent household income, educational attainment, marital status, living status, driving status, frequency of going out, land value, and duration of walking.

Table 3. Sensitivity analysis stratified by latitude

Model 1 controlled for sex, age, education, equivalent household income, marital status, living situation, driving status, land value, and population density.

Model 2 controlled for sex, age, education, equivalent household income, marital status, living situation, driving status, land value, population density, frequency of going out, and duration of walking time.

Model 3 controlled for sex, age, education, equivalent household income, marital status, living situation, driving status, land value, population density, frequency of seeing friends, and social participation.

Table 4. Sensitivity analysis stratified by population density

Model 1 controlled for sex, age, education, equivalent household income, marital status, living situation, driving status, and land value.

Model 2 controlled for sex, age, education, equivalent household income, marital status, living situation, driving status, land value, frequency of going out, and duration of walking time.

Model 3 controlled for sex, age, education, equivalent household income, marital status, living situation, driving status, land value, frequency of seeing friends, and social participation.

* We adjusted the connectivity criteria in urban and rural areas based on population density.

Table 5. Sensitivity analysis stratified by gender

Model 1 controlled for age, education, equivalent household income, marital status, living situation, driving status, and land value.

Model 2 controlled for age, education, equivalent household income, marital status, living situation, driving status, land value, frequency of going out, and duration of walking time.

Model 3 controlled for age, education, equivalent household income, marital status, living situation, driving status, land value, frequency of seeing friends, and social participation.

Table 6-1. Using Quintile and Continuous Value of Street Connectivity as Explanatory Variables

(Exposure: Intersection density)

| Quintile of Intersection density | Model 1 | Model 2 | Model 3 |
| --- | --- | --- | --- |
|  | OR(95% CI) | OR(95% CI) | OR(95% CI) |
| Lowest (≤116) | Ref. | | |
| Low (117–169) | 0.88(0.77–1.02) | 0.89(0.77–1.03) | 0.89(0.77–1.03) |
| Moderate (170–204) | 0.95(0.81-1.11) | 0.95(0.81-1.12) | 0.97(0.83-1.14) |
| High (205–238) | 0.87(0.73-1.03) | 0.86(0.72-1.02) | 0.87(0.74-1.04) |
| Highest (>238) | **0.77(0.64–0.93)** | **0.76(0.63–0.92)** | **0.77(0.64–0.93)** |
| Continuous value of intersection density | Model 1 | Model 2 | Model 3 |
|  | OR(95% CI) | OR(95% CI) | OR(95% CI) |
| Intersection density | **0.99(0.99–0.99)** | **0.99(0.99–0.99)** | **0.99(0.99–0.99)** |

Ref. = Reference group; OR = Odds ratio; 95% CI = 95% Confidence interval;
*p*-Value = significant p-values (<0.05) are in bold

Table 6-2. Using Quintile and Continuous Value of Street Connectivity as Explanatory Variables

(Exposure: Space syntax connectivity)

| Quintile of space syntax connectivity | Model 1 | Model 2 | Model 3 |
| --- | --- | --- | --- |
|  | OR(95% CI) | OR(95% CI) | OR(95% CI) |
| Lowest (<2.5) | Ref. | | |
| Low (2.5–2.7) | 0.95(0.82–1.09) | 0.94(0.82–1.09) | 0.95(0.82–1.10) |
| Moderate (2.7–2.9) | **0.81(0.70-0.94)** | **0.81(0.70-0.95)** | **0.82(0.70-0.95)** |
| High (2.9–3.3) | **0.85(0.73-0.99)** | **0.85(0.73-0.99)** | **0.85(0.72-0.99)** |
| Highest (>3.3) | 0.89(0.75–1.06) | 0.88(0.74–1.05) | 0.88(0.74–1.05) |
| Continuous value of space syntax connectivity | Model 1 | Model 2 | Model 3 |
|  | OR(95% CI) | OR(95% CI) | OR(95% CI) |
| Space syntax connectivity | 0.92(0.81–1.04) | 0.91(0.80–1.03) | 0.91(0.80–1.03) |

Ref. = Reference group; OR = Odds ratio; 95% CI = 95% Confidence interval;
*p*-Value = significant p-values (<0.05) are in bold
